# *FRIZZLED7* Is Required for Tumor Inititation and Metastatic Growth of Melanoma Cells

**DOI:** 10.1371/journal.pone.0147638

**Published:** 2016-01-25

**Authors:** Shweta Tiwary, Lei Xu

**Affiliations:** Department of Biomedical Genetics, University of Rochester Medical Center, Rochester, New York,14642, United States of America; Istituto Superiore di Sanità, ITALY

## Abstract

Metastases are thought to arise from cancer stem cells and their tumor initiating abilities are required for the establishment of metastases. Nevertheless, in metastatic melanoma, the nature of cancer stem cells is under debate and their contribution to metastasis formation remains unknown. Using an experimental metastasis model, we discovered that high levels of the WNT receptor, FZD7, correlated with enhanced metastatic potentials of melanoma cell lines. Knocking down of *FZD7* in a panel of four melanoma cell lines led to a significant reduction in lung metastases in animal models, arguing that FZD7 plays a causal role during metastasis formation. Notably, limiting dilution analyses revealed that *FZD7* is essential for the tumor initiation of melanoma cells and *FZD7* knockdown impeded the early expansion of metastatic melanoma cells shortly after seeding, in accordance with the view that tumor initiating ability of cancer cells is required for metastasis formation. FZD7 activated JNK in melanoma cell lines *in vitro* and the expression of a dominant negative JNK suppressed metastasis formation *in vivo*, suggesting that FZD7 may promote metastatic growth of melanoma cells via activation of JNK. Taken together, our findings uncovered a signaling pathway that regulates the tumor initiation of melanoma cells and contributes to metastasis formation in melanoma.

## Introduction

Melanoma is curable with a 5 year survival rate of >97% if detected early. Once metastatic, the 5-year survival drops to ~15% when treated with conventional therapies [1]. Sixty-percent of melanomas express a constitutively active form of BRAF (BRAF^CA^), that is inhibited by BRAF^CA^-specific inhibitors (BIs). The BIs are potent therapeutic agents for melanoma treatment, but resistance develops quickly in treated patients [2, 3]. Furthermore, BIs are not effective against the ~40% of melanomas that express wild-type BRAF. Alternative or combinatory strategies are needed to improve the treatment of metastatic melanoma, especially those that do not respond to BIs. An in-depth understanding of melanoma biology and metastasis would greatly facilitate the effort in this area.

Metastasis is a multi-step process. Cancer cells must detach from the primary tumor, intravasate into circulation, survive in circulation, extravasate (exit) from the circulation, and survive/grow in a distant organ (defined as metastasis growth) to successfully metastasize [4, 5]. The last step, metastasis growth, is considered a rate-limiting step during metastasis. Both experimental and clinical data showed that metastases are relatively rare compared with the number of circulating tumor cells [5]. Furthermore, metastatic cells frequently remain dormant for months or years before giving rise to detectable metastases [6].

One key process during metastatic growth is the initiation of growth of extravasated cancer cells, exemplified by their survival and limited expansion into micrometastases in a distant organ. This process resembles tumor initiation from cancer stem cells in primary tumors and, as such, metastatic cells are thought to possess cancer stem cell properties [7]. Nevertheless, the existence of a distinct population of cancer stem cells in melanoma is controversial. Some reported that the majority of cancer cells in a human melanoma have tumor initiating potentials [8], whereas others observed subpopulations of melanoma cells that have enhanced tumor-initiating abilities than the bulk of tumor [9, 10] or are required for continuous tumor maintenance [11]. The connection between cancer stem cells and metastasis formation for metastatic melanoma remains an open question.

We use an experimental metastasis model to investigate the underlying mechanisms of metastatic growth in melanoma. A poorly metastatic melanoma cell line (A375P) was injected intravenously into immunodeficient mice. Lung metastases were isolated and derived into cell lines, which had enhanced metastatic potential than the parental line [12]. Bioinformatic analyses identified a set of genes that were either up- or down-regulated in the highly metastatic derivatives compared with the poorly metastatic parental line. One of the up-regulated genes was *FZD7*. FZD7 (Frizzled-7) is a receptor of WNT (Wingless) proteins [13], which stimulate either a canonical signaling pathway via β-catenin, or a G protein-coupled signaling or planar cell polarity pathway [14, 15]. The WNT signaling is implicated extensively in stem cell maintenance and differentiation, and FZD7 has been shown to regulate stem cell functions in a variety of normal tissues and cancer types [16–23]. We report here that FZD7 is required for tumor initiation from melanoma cell lines, revealing for the first time a signaling pathway that regulates the cancer stem cell property in melanoma. Consistent with its function in tumor initiation, knocking down FZD7 led to an inhibition of growth initiation of metastatic melanoma cells after seeding in lung. These findings are consistent with the connection between initiation (or cancer stem cells) and metastatic growth in melanoma. Finally, FZD7 activates JNK in melanoma cells *in vitro*, and the expression of a dominant negative JNK inhibited their metastasis formation *in vivo*, suggesting that FZD7 promotes melanoma metastasis by activating JNK.

## Results

### *FZD7* is required for metastasis formation of melanoma cell lines irrespective of their BRAF mutation status or BI sensitivity

**Fig 1:**
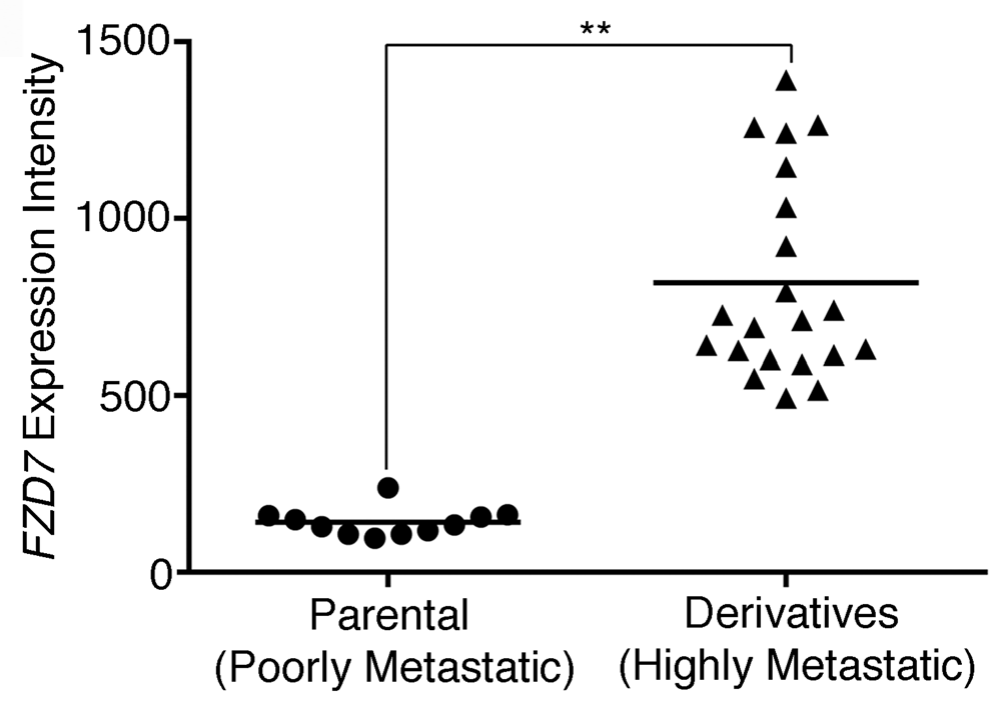
*FZD7* expression correlates with melanoma malignancy. Expresson values of *FZD7* mRNA in tumor samples from highly metastatic derivatives and those from the poorly metastatic parental line. **: Mann-Whitney test, *p* < 0.01. (n = 11 for parental, n = 21 for metastatic derivatives).

**Fig 2:**
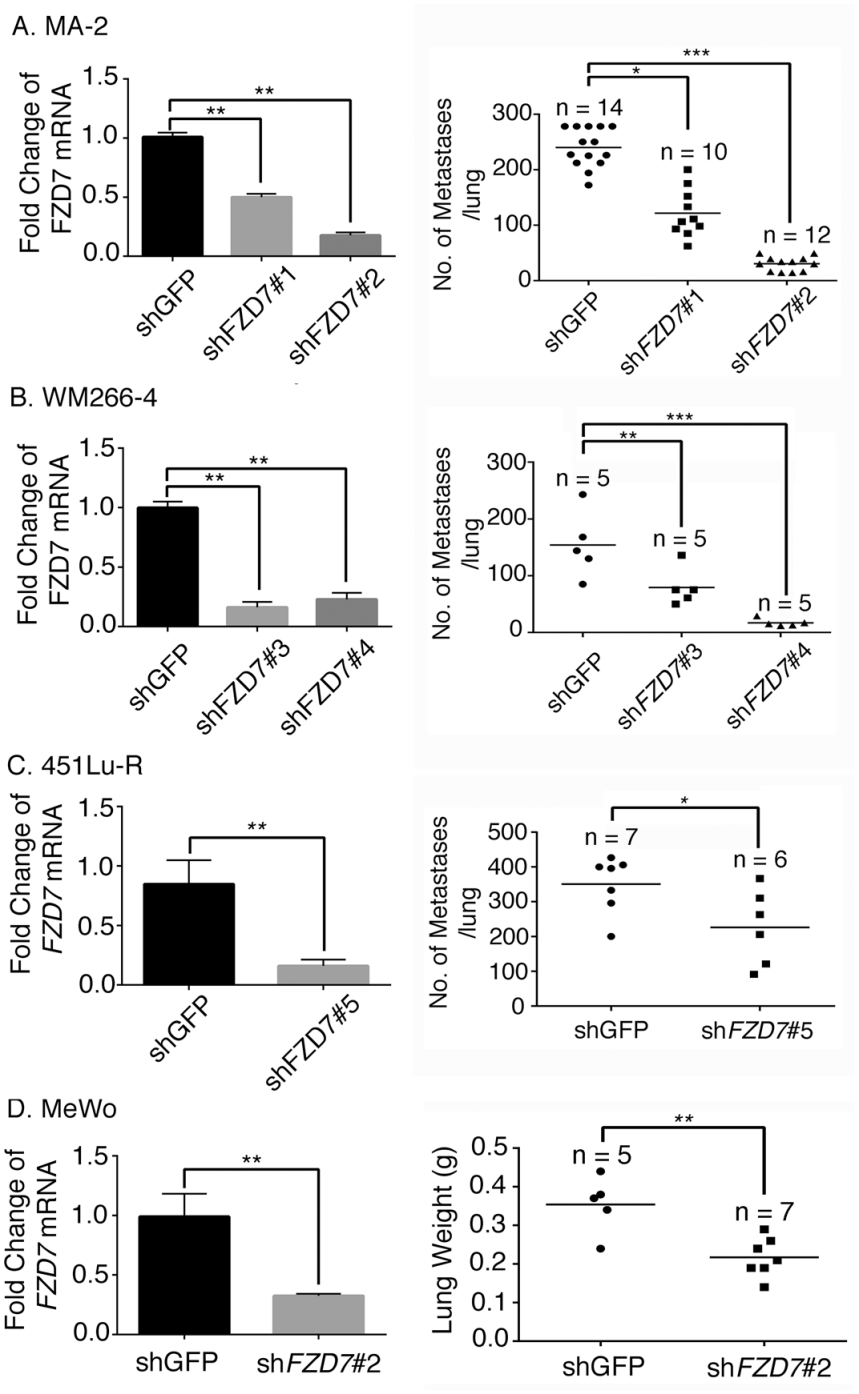
*FZD7* knockdown reduces metastasis potential of multiple melanoma cell lines. *FZD7* was knocked down by shRNA in MA-2 (A), WM266-4 (B), 451Lu-R (C) and MeWo (D) cell lines. The extent of knockdown was measured by qRT-PCR (left panels). The knockdown cell lines and the shGFP control were injected intravenously into NSG mice. The number of metastases formed in lung was counted (right panels). Student’s t test, *: *p* < 0.05; **: *p* < 0.01; ***: *p* < 0.001.

Using the experimental metastasis model, we derived highly metastatic melanoma cell lines from a poorly metastatic parental line, A375P [12]. Gene expression analyses showed that *FZD7*, the gene encoding a WNT receptor, was highly up-regulated in tumor samples from the highly metastatic derivatives relative to those from the parental line (Fig 1). We then investigated whether FZD7 plays a causal role in melanoma metastasis, using the experimental metastasis model. Four melanoma cell lines were used for our analyses (Table 1). They carry either BRAF^CA^ mutation (MA-2 and WM266-4) [24], express wild-type BRAF (MeWo) [25], or express BRAF^CA^ but have gained resistance to BIs via *in vitro* selection (451Lu-R) [26]. In each cell line, *FZD7* was knocked down by shRNA(s) and the efficiency of knockdown was measured by qRT-PCR. A two to four fold reduction was achieved (Fig 2A–2D, left panels). The knockdown lines and the controls expressing an shRNA against *GFP* were injected intravenously into the immunodeficient NSG mice. A significant reduction in lung metastases was observed in all the knockdown cell lines (Fig 2A–2D, right panels), demonstrating that *FZD7* is required for metastasis formation in melanomas. These four melanoma cell lines carry different mutations and/or exhibit different sensitivities to BIs (Table 1) and therefore represent some of the heterogeneity observed in human melanomas. Regulation of metastasis by FZD7 in all four lines suggest that this may be a shared mechanism among metastatic melanomas.

**Table 1:** BRAF mutation status and BI sensitivity of melanoma cells.

Melanoma cell line	BRAF Mutation status	BRAF inhibitor sensitivity
MA-2	V600E	Sensitive
WM266-4	V600D	Sensitive
451Lu-R	V600E	Resistant
MeWo	WT	Resistant

### *FZD7* is required for tumor initiation of melanoma cells *in vivo*

**Fig 3:**
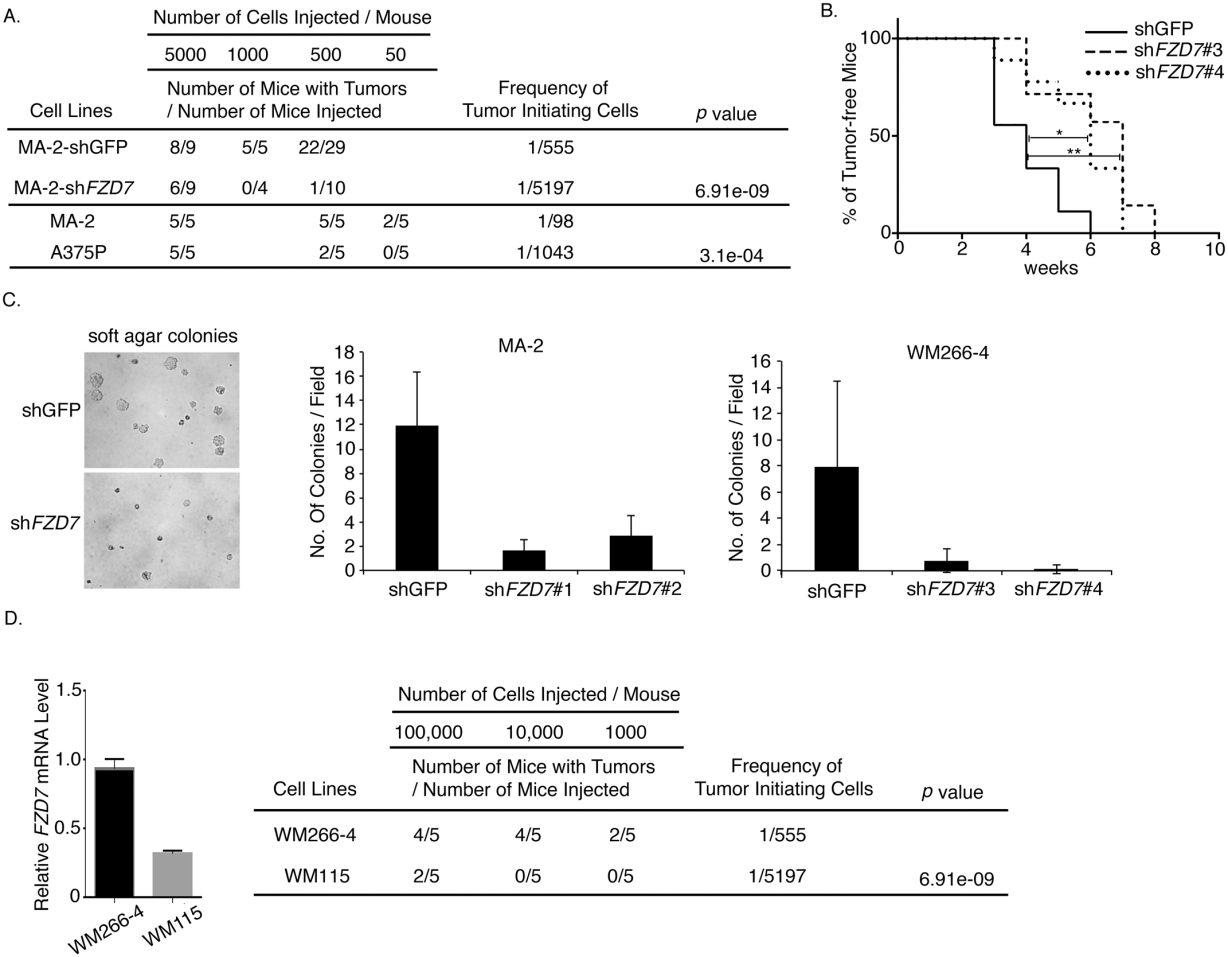
*FZD7* is required for tumor initiation of melanoma cells. A. Limiting dilution assays for MA-2(shGFP) and MA-2(shFZD7), or A375P and MA-2 cells. Tumor incidence was recorded 12 weeks after inoculation of the cells. B. Tumor-onset analyses on WM266-4(shGFP) or WM266-4(shFZD7) cells. The percent of tumor-free mice over time is shown. Total number of mice analyzed was 9 for WM266-4(GFP), 7 for WM266-4(shFZD7#3), and 9 for WM266-4(shFZD7#4) cells. Log-rank test, *: *p* < 0.05; **: *p* < 0.01. C. *FZD7* knockdown in MA-2 and WM266-4 cells led to reduced number of colonies in soft agar. Left: images of colonies in soft agar. Middle and right: the average number of colonies per field from control or FZD7-knockdown MA-2 or MeWo cells. D. Left: relative levels of *FZD7* mRNA in WM266-4 and WM115 cells measured by qRT-PCR. Right: limiting dilution assays for WM266-4 and WM115 cells.

Because of the known function of WNT signaling and FZD7 in stem cell maintenance and differentiation, we investigated whether FZD7 plays roles in melanoma stem cells. Limiting dilution assay, a standard approach to determine the tumor initiation ability of cancer cells [8], was performed. Different numbers of *FZD7*-knockdown and control MA-2 cells (5000, 1000, or 500 cells/mouse) were injected subcutaneously into NSG mice. Tumor occurrence was recorded 12 weeks after injections. *FZD7* knockdown in MA-2 cells led to a significant reduction in tumor incidence (Fig 3A), suggesting that FZD7 is essential for tumor initiation of MA-2 cells. A similar reduction in tumor initiation was observed in WM266-4 cells expressing *FZD7* shRNA (Fig 3B), although in this case the knockdown cells eventually grew into tumors in all the mice injected. Analysis of the tumors showed that they had all escaped knockdown and regained expression of *FZD7* (S1 Fig). Finally, the effects of FZD7 on tumor initiation were recapitulated *in vitro* by soft agar colony formation assay (see Materials and Methods). Knocking down of *FZD7* led to a significant reduction in colony formation from both MA-2 and WM266-4 cell lines (Fig 3C). These data collectively demonstrate that FZD7 is required for tumor initiation of melanoma cells *in vivo*.

Since high levels of *FZD7* expression correlate with metastatic potential of melanoma cell lines (Fig 1), we asked whether this correlation is linked to its function on tumor initiation. Pairs of human melanoma cell lines that share an ancestry but exhibit different metastatic potentials were utilized to address this issue. One pair is MA-2/A375P. The highly metastatic MA-2 cell line was derived from the poorly metastatic A375P cells [12] and expresses a higher level of *FZD7* (Fig 1). Limiting dilution assays showed that the tumor initiating frequency of MA-2 cells was significantly higher than that of A375P cells (Fig 3A), consistent with a link between FZD7-mediated tumor initiation and metastatic potential. Another pair of melanoma cell lines was WM266-4/WM115. They were derived from melanomas of the same patient, but WM115 was from the primary tumor and WM266-4 was from a metastasis. The WM266-4 cells expressed higher levels of *FZD7* mRNA than WM115 cells (Fig 3D, left) and had a higher tumor initiating frequency (Fig 3D, right), further supporting the association among *FZD7* expression, tumor initiation, and melanoma metastasis in patients.

### FZD7 is required for the initiation of metastatic growth of melanoma cells

**Fig 4:**
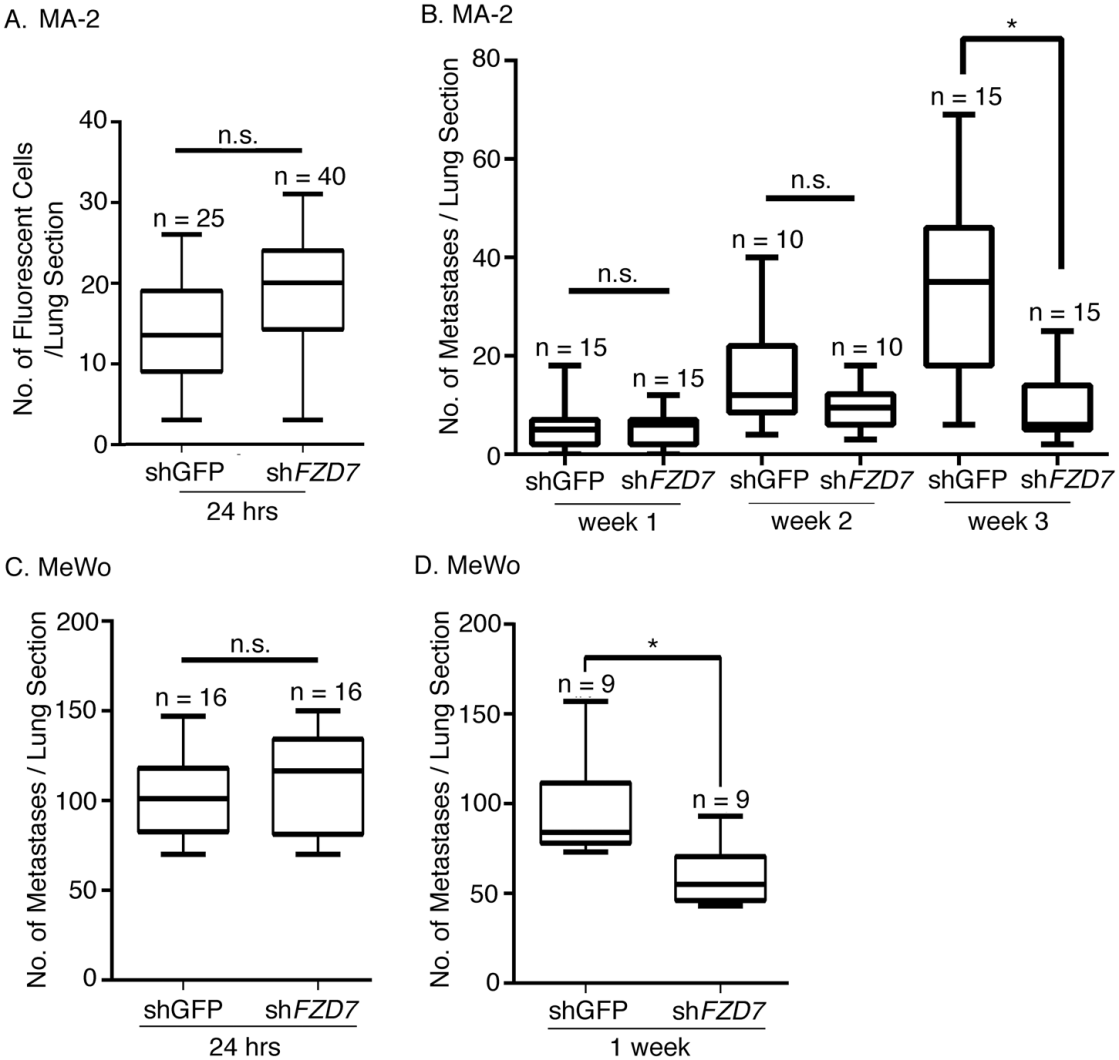
Time course of FZD7 function during melanoma metastasis. Control or *FZD7*-knockdown MA-2 or MeWo cells were injected into NSG mice. Lungs were harvested 24 hrs (n = 5 for MA-2(shGFP), n = 8 for MA-2(shFZD7), n = 4 for MeWo(shGFP), n = 4 for MeWo(shFZD7)), 1 week (n = 6 for MA-2(shGFP), n = 6 for MA-2(shFZD7), n = 4 for MeWo(shGFP), n = 4 for MeWo(shFZD7), 2 weeks (n = 4 for MA-2(shGFP), n = 6 for MA-2(shFZD7)), or 3 weeks (n = 3 for MA-2(shGFP), n = 3 for MA-2 (shFZD7)) after injections. ~3–5 sections per lung were stained and metastases counted. No difference in the number of metastases was observed between control and knockdown at the 24 hr time point for both cell lines (A, C). For MA-2 cells, *FZD7* knockdown led to a reduction in metastases three weeks after injections (B). *: Mann-Whitney test, *p* < 0.05. For MeWo cells, *FZD7* knockdown led to a reduction in metastases one week after injections (D). *: Mann-Whitney test, *p* < 0.05.

We then investigated whether the effects of FZD7 on tumor initiation contribute to its function on metastasis initiation in melanoma, using the experimental metastasis model. The experimental metastasis model analyzes the process of metastasis after cancer cells disseminate into the circulation [4]. These disseminated cancer cells are thought to arrest in blood vessels within minutes after entering the circulation, extravasate within hours, and survive and grow for days, weeks, to years before forming macroscopically detectable metastases [27]. To investigate at which point(s) FZD7 comes into play, lungs from mice injected with *FZD7*-knockdown or control MA-2 and MeWo cells were harvested 24 hrs, one week, two weeks, or three weeks after injection, and sectioned. Metastases were either visualized by fluorescence imaging if cells were pre-loaded with the green fluorescent cell tracker dye, CMFDA [28], or by immunohistochemical analyses using an antibody specific to human vimentin (see Materials and Methods). Knocking down of *FZD7* did not reduce the number of metastases from either MA-2 (Fig 4A) or MeWo cells 24 hrs after injections (Fig 4C), indicating that FZD7 is not required for the seeding and initial survival of melanoma cells in lung. In fact, for MA-2 cells, the effect of FZD7 on the number of metastases was not observed until 3 weeks post injection (Fig 4B, right). For MeWo cells, this effect was observed earlier, at 1 week after injections (Fig 4D).

**Fig 5:**
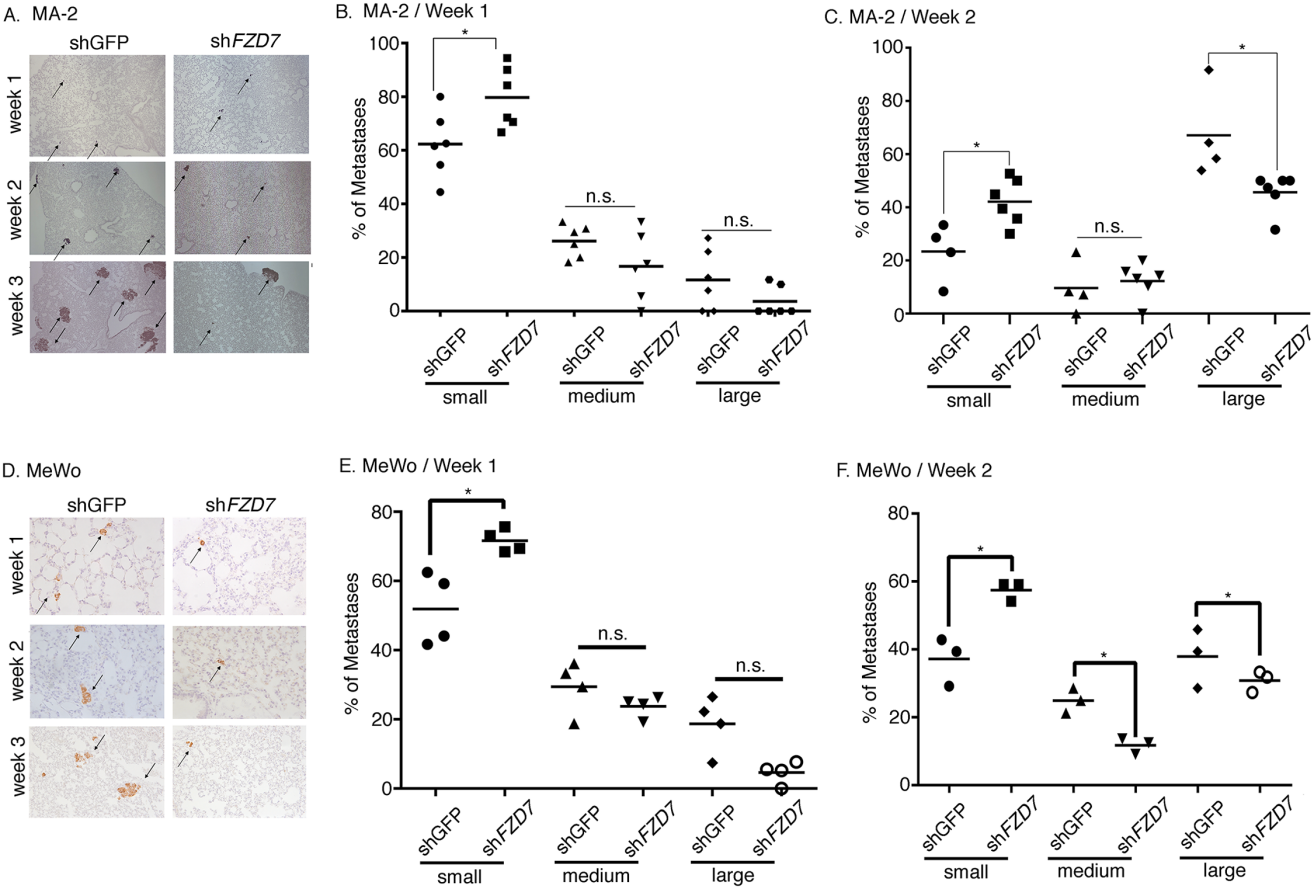
*FZD7* regulates the size of metastases from MA-2 and MeWo Cells. A. Week1, week2, and week 3 metastases (arrows) from MA-2(shGFP) and MA-2(sh*FZD7*) cells were stained with the antibody against human vimentin (purple). B. Size distribution of metastases from MA-2(shGFP) and MA-2(sh*FZD7*) cells 1 week post injection. *: Student’s t test, *p* < 0.05. C. Size distribution of metastases from MA-2(shGFP) and MA-2(sh*FZD7*) cells 2 weeks post injection. *: Student’s t test, *p* < 0.05. D. Week1 and week2 metastases (arrows) from MeWo(shGFP) and MeWo(sh*FZD7*) cells were stained with the antibody against human vimentin as in A. E. Size distribution of metastases from MeWo(shGFP) and MeWo(sh*FZD7*) cells 1 week post injection. *: Mann-Whitney test, *p* < 0.05. F. Size distribution of metastases from MeWo(shGFP) and MeWo(sh*FZD7*) cells 2 weeks post injection. *: Mann-Whitney test, *p* < 0.05.

Although the difference in metastasis number was not observed until 3 weeks post injection for MA-2 (sh*FZD7*) and control samples, a marked difference in the size of metastases was noted as early as one week after injection (Fig 5A). To quantitate this size difference, metastases were divided into small, medium, and large groups, based on the number of cells each of them contains (see Materials and Methods for details). At one week post injection, a higher portion of MA-2(sh*FZD7*) metastases were in the small group compared with controls, although the portion of medium or large metastases did not differ (Fig 5B). This finding indicates that FZD7 is required for the initiation of growth of MA-2 cells after their seeding in lung. At week 2, however, metastases from MA-2(sh*FZD7*) cells were not only enriched for small metastases, but were deficient in large metastases, compared with the control (Fig 5C). This indicates that the defect in growth initiation of MA-2(sh*FZD7*) cells led to a decrease in the number of large metastases later on, although the total number of metastases was still not affected until one week later (Fig 4B, week 3). Similar to observations from MA-2 cells, *FZD7* knockdown in MeWo cells resulted in the enrichment for small metastases at the week 1 time point (Fig 5D–5F), and both enrichment of small metastases and deficiency in medium and large metastases at the week 2 time point (Fig 5F).

### FZD7 is required for the proliferation of melanoma cells during metastatic growth

**Fig 6:**
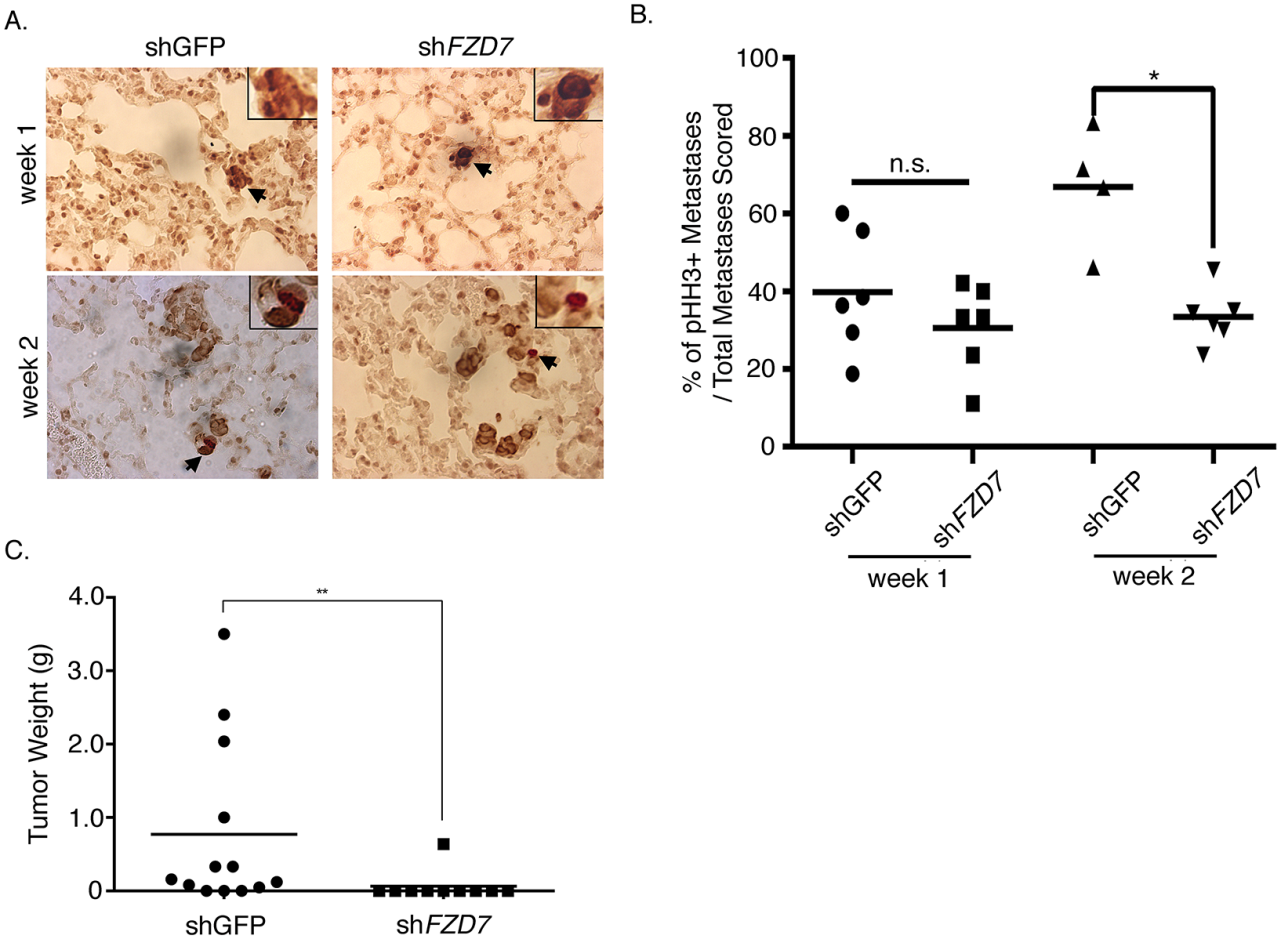
*FZD7* is required for cell proliferation in metastases from MA-2 cells. A. Immunostaining of metastases from MA-2(shGFP) or MA-2(sh*FZD7*) cells with the antibodies against phospho-histone H3 (dark red) and human vimentin (purple). Inset shows the high magnification of images pointed by black arrows. B. The percentage of pHH3+ metastases over the total number of metastases scored at week-1 (n = 6 for MA-2(shGFP) or MA-2(shFZD7) samples) or week-2 (n = 4 for MA-2(shGFP) and n = 6 for MA-2(shFZD7) samples) post injection. *: Student’s t test, *p* < 0.05. C. Subcutaneous tumor growth of MC-1(shGFP) (n = 13) and MC-1(sh*FZD7*) (n = 10) cells. **: Student’s t test, *p* < 0.01.

The observed effects of FZD7 on early expansion of metastasis led us to ask whether FZD7 regulates the proliferation of metastatic cells *in vivo*. Lung sections from mice injected with *FZD7* knockdown or control MA-2 cells were stained with the anti-phospho-Histone H3 (pHH3) antibody (to label cells at metaphase) and the anti-human vimentin antibody (to label tumor cells) (Fig 6A). By week 1, the portion of pHH3-positive metastases did not differ between the *FZD7*-knockdown and control samples (Fig 6B). This was unexpected, given that at this stage the size of knockdown metastases was significantly smaller than controls (Fig 5). By week 2, however, a reduction in pHH3+ metastases was observed in the knockdown group (Fig 6B), suggesting that *FZD7* is required for the proliferation of melanoma cells in lung. Further supporting its function on cell proliferation, subcutaneous growth of MA-2(sh*FZD7*) cells was compromised relative to the control (Fig 6C). Notably, the proliferation of *FZD7*-knockdown cells did not differ from the control cells *in vitro* (S2 Fig), so the effects of *FZD7* on cell proliferation are not intrinsic to the cells but depend on the microenvironment *in vivo*.

### FZD7 signals through the non-canonical WNT pathway and activates JNK

**Fig 7:**
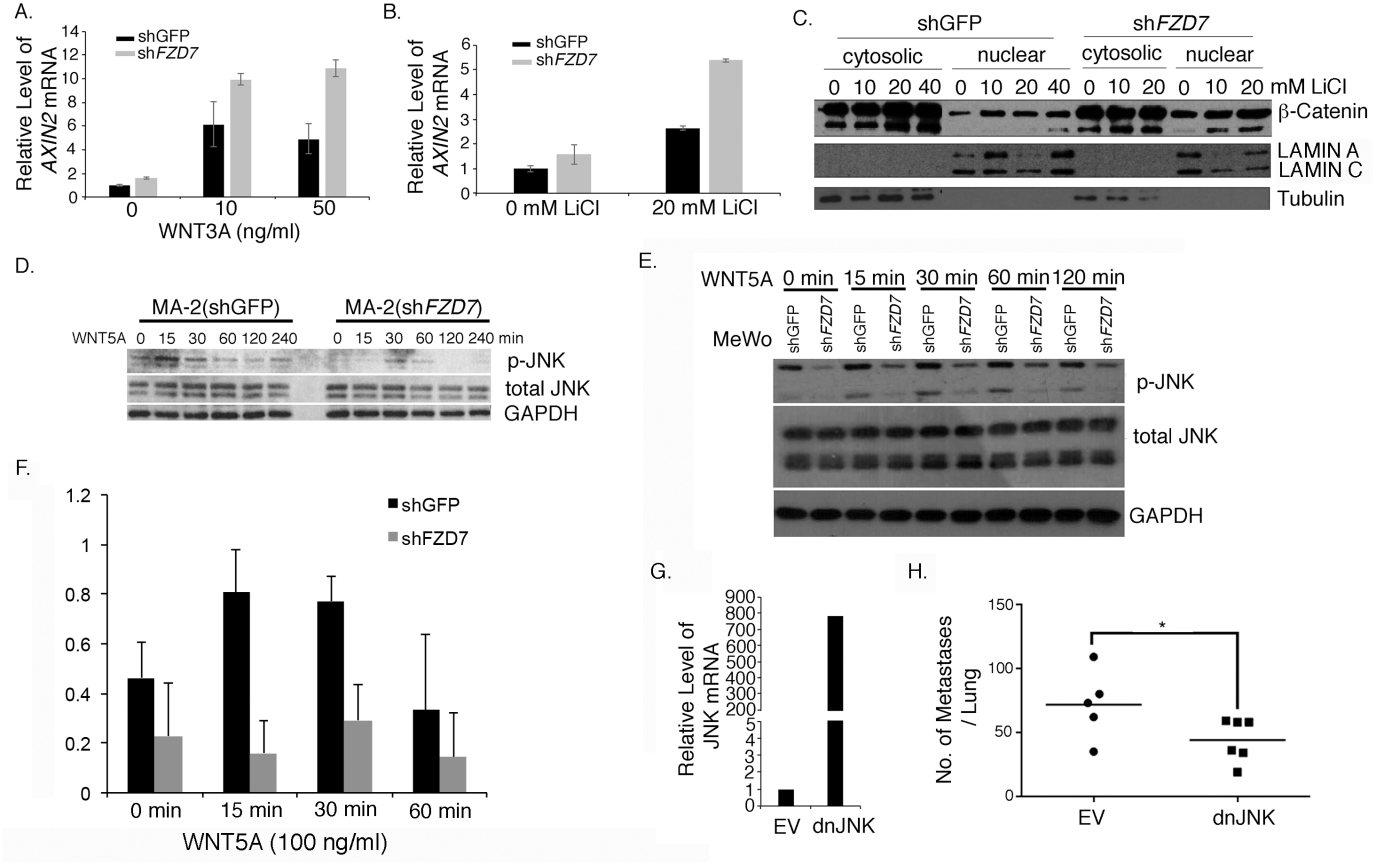
FZD7 promotes melanoma metastasis via activation of JNK. A. WNT3A treatment of MA-2(shGFP) and MA-2(sh*FZD7*) cells led to an increase in AXIN2 mRNA in both cell lines, but the level of AXIN2 mRNA was higher in the knockdown cells across all treatments. B. LiCl treatment of MA-2(shGFP) and MA-2(sh*FZD7*) cells led to an increase in AXIN2 mRNA in both cell lines, but the increase was more prominent in the knockdown cells. C. The cytosolic and nuclear fractions from LiCl-treated cells were probed with the anti-β-catenin antibody. Lamin and tubulin were used as the loading control for each fraction. D. MA-2(shGFP) and MA-2(sh*FZD7*) were treated with WNT5A and probed with antibodies against p-JNK and total JNK. GAPDH was used as a loading control. E. MeWo(shGFP) and MeWo(sh*FZD7*) were treated with WNT5A and probed with antibodies against p-JNK and total JNK. GAPDH was used as a loading control. F. The ratio between p-JNK and total JNK on western blots represented in D and E. G. qRT-PCR analyses to measure the over-expression of dnJNK in MA-2 cells. H. MA-2(EV) and MA-2(dnJNK) cells were injected intravenously into NSG mice. A significant reduction in metastasis formation was observed in dnJNK-overexpressing cells. *: Mann-Whitney test, *p* < 0.05.

FZD7 is a WNT receptor and is capable of signaling through either canonical or non-canonical WNT signaling pathways [29], both of which have been implicated in melanoma progression [30, 31]. It was not clear which of these pathways mediates FZD7 function during melanoma metastasis. FZD7 has been shown to interact with WNT3A [32], WNT5A [33, 34], WNT8 [35], and WNT11 [36]. We retrieved the mRNA expression values of these ligands from the microarray data on human melanoma metastases [12]. *WNT3* and *WNT5A* were detected at a much higher level than *WNT8* and *WNT11* (S3 Fig), so we investigated further whether WNT3 and WNT5A signal through FZD7 in melanoma cells. WNT3 is a typical canonical WNT ligand [35] and induces nuclear translocation of β-catenin and its target gene expression, such as *AXIN2*. Treating MA-2(shGFP) or MA-2(sh*FZD7*) cells with 0, 10 ng/ml, or 30 ng/ml of WNT3A resulted in the induction of *AXIN2* mRNA, as expected (Fig 7A). However, the *AXIN2* level was higher in *FZD7*-knockdown cells compared with the control across all treatments (Fig 7A), suggesting that FZD7 not only does not stimulate canonical WNT signaling in MA-2 cells but may suppress it. This suppression of canonical WNT signaling by FZD7 was confirmed by analyses using LiCl. LiCl inhibits GSK3 and so stablizes β-catenin and constitutively activates canonical WNT signaling [37]. Its treatment resulted in an elevated induction of *AXIN2* in both control and *FZD7*-knockdown cells, but the increment in knockdown cells was more prominent (Fig 7B). Along the same line, nuclear accumulation of β-catenin was observed in LiCl-treated control and knockdown cells but the accumulation was more pronounced in the knockdown cells (Fig 7C), confirming the suppressive role of FZD7 on canonical WNT signaling.

In contrast to WNT3, WNT5A is thought to induce non-canonical WNT signaling by activating JNK. It is frequently up-regulated in advanced melanoma and has been reported to promote melanoma metastasis [38]. Its metastasis-promoting function had not been associated with FZD7, although its’ interaction with FZD7 has been reported [33, 34, 39]. To analyze whether WNT5A activates FZD7 in melanoma cells, we treated MA-2(shGFP) and MA-2(sh*FZD7*) cells, or MeWo(shGFP) and MeWo(sh*FZD7*) cells with recombinant WNT5A. The level of phosphorylated JNK in treated cells was examined. *FZD7*-knockdown led to attenuated phosphorylation of JNK (Fig 7D–7F) in both knockdown cell lines, suggesting that FZD7 trasmits non-canonical WNT signaling from WNT5A in melanoma cells. We also noticed that in the absence of added WNT5A phospho-JNK was detected in both lines and its level was dependent on FZD7 expression (Fig 7D–7F, 0 min). We reasoned that these cells must express endogenous WNT5A or other non-canonical WNT ligands, which activate JNK via FZD7 in a cell autonomous manner. Indeed, qRT-PCR analyses show that *WNT5A* is expressed in MA-2 and A375P cell lines, as well as in subcutaneous tumors or lung metastases from MA-2 cells (S4 Fig). *WNT5A* is also expressed in mouse lung, but at a much lower level than the lung metastases from MA-2 cells, indicating that the majority of WNT5A in the metastases is produced by MA-2 cells and consequently acts cell autonomously. On the contrary, in MeWo cells *WNT5A* was undetectable (S4 Fig), arguing that a different WNT ligand must be expressed in these cells to induce autocrine signaling via FZD7 and p-JNK.

JNK is a stress-response MAP kinase [40]. It has been implicated in cancer progression but its function is complex and context dependent [41] (see Discussion). The function of JNK on melanoma metastasis is not understood. *In vitro* activation of JNK by FZD7 suggests that FZD7 might promote melanoma metastasis via JNK. To test this, a dominant negative mutant of JNK [42] was expressed in MA-2 cells (Fig 7G). It significantly impeded metastasis formation from these cells (Fig 7H), supporting the essential role of JNK on FZD7-mediated metastatic growth of melanoma.

## Discussion

The existence and characteristics of cancer stem cells in melanoma are outstanding questions and their connection with metastasis formation has not been investigated. We report here that a WNT receptor, FZD7, is required for both tumor initiation of melanoma cells and their metastatic growth in lung. These findings suggest that FZD7-mediated signaling may be an essential component of cancer stem cell property in melanoma, and that melanoma stem cells may contribute to metastasis formation, similar to observations in other cancer types. The shared *FZD7*-dependence among metastases from BI-sensitive and–resistant melanoma cells strongly indicate that these functions of FZD7 may be clinically relevant and targeted for melanoma treatment. Our studies, however, were all performed in the experimental metastasis model, which is useful to dissect mechanisms of metastasis but does not recapitulate the full metastasis process in human. Future studies using alternative models, such as transgenic tumor models, will be needed to further establish its function in melanoma metastasis.

Tumor initiation is required for metastasis, so disseminated cancer cells that give rise to metastases are assumed to be cancer stem cells, although this had not been established for melanoma. After seeding in a distant organ, metastatic cancer cells typically go through a period of dormancy before the onset of proliferation [43, 44]. Dormancy is defined as a state at which cells neither proliferate nor go through apoptosis. In the experimental metastasis model, most melanoma cells stay at this phase within the first week after seeding (unpublished observation). These cells are slow cycling and thought to exhibit stem cell properties [44]. Their reactivation would model the tumor initiation from cancer stem cells. Our data show that knocking down of FZD7 led to a decrease in metastasis size at this stage, consistent with its function on tumor initiation of melanoma cells. The lack of difference in pHH3 staining between control and FZD7-knockdown metastases at this stage (Fig 6B), despite their size difference, may be due to the slow-cycling nature of tumor initiating cells.

Tumor initiating cells (or cancer stem cells) typically represent a distinct population of cells that undergo self-renewal and differentiation to give rise to remaining cells in a tumor. In most cancer types, these cells are rare compared with the bulk of the tumor. It had been hotly debated, however, whether this type of cancer stem cells exists in melanoma. Quintana et al elegantly showed that the majority of cancer cells in a human melanoma could have tumor initiating potentials [8], whereas others reported distinct subpopulations of melanoma cells that exhibit enhanced tumor-initiating abilities than the bulk of tumor [9, 10] or are required for continuous tumor maintenance [11]. Despite these differences, the causal roles of FZD7 on tumor initiation, as reported here, indicate that the tumor initiating properties of melanoma cells are subject to similar regulations that impose on stem cells from other cancer types. FZD7-mediated WNT signaling would be an example. FZD7 has been shown to play essential functions in stem cell maintenance from multiple tissues and cancer types [16–23] and WNT signaling is well known to regulate stem cell properties.

Nevertheless, our findings do not differentiate the function of FZD7 on tumor initiation from its function on cell proliferation in the bulk of metastases during metastatic growth. This is distinct from conventional view on cancer stem cells, in which tumor initiation is a separate entity from cell proliferation in the bulk of tumor [45]. It is possible that the tumor initiation of melanoma stem cells and the proliferation potential in the bulk of melanomas are regulated by shared mechanisms, which might explain the prevalence of stem cells observed in melanoma [8]. However, this does not necessarily suggest that all melanoma cells in a tumor are equivalent. It is apparent that subpopulations of melanoma cells with distinct tumor initiating and tumor maintenance potential could be identified from the bulk of tumor [9–11]. Likely, the tumor initiating property and other characteristics (such as cell proliferation, tumor maintenance) of melanoma cells are regulated by both shared and distinct mechanisms. FZD7-mediated signaling would be one of the shared mechanisms. Other pathways might regulate only one but not the other process.

Both canonical and non-canonical WNT signaling are implicated in stem cell regulation and metastasis. *FZD7* could mediate both [29]. In cancer, the function of FZD7 has mostly been attributed to canonical WNT signaling, although some evidence on non-canonical signaling has been presented. The effects of canonical WNT signaling on melanoma progression are controversial [31]. Some reports support its function on promoting melanoma progression but others reported its association with reduced cell proliferation and improved survival and prognosis of melanoma patients. Our data suggest that FZD7 activates JNK through non-canonical signaling to support metastatic growth in melanoma. JNK is best known as a stress response MAPK [40]. Its function in cancer is complex and context dependent [41]. In melanoma, JNK activity is reportedly elevated upon drug treatment and thought to cooperate with drug-induced cell death [46]. Consequently, JNK inhibitors may not be used in combination with other therapies. Under unstressed conditions, however, JNK appears to promote melanoma development. For example, phosphorylation of JNK was found to correlate with a shorter disease-free survival of patients with superficial spreading melanomas [47], and JNK inhibitors blocked cell proliferation *in vitro* and *in vivo* [48]. Our data show for the first time that JNK activity promotes metastatic growth of melanoma cells. JNK inhibitors may thus have therapeutic value for treating metastatic melanoma, although they may not be used in combination with or following other types of treatments.

## Materials and Methods

### Mice, cell lines, and reagents

The NSG mice (NOD.Cg-*Prkdc*^*scid*^ *Il2rg*^*tm1Wjl*^/SzJ, the Jackson Laboratory) were used in this study, and were provided by a co-op core facility at University of Rochester Medical Center [49]. All mice were housed in the animal facility at the University of Rochester Medical Center, in strict accordance to the Guide for the Care and Use of Laboratory Animals of the USPHS, the USDA Animal Welfare act, and the Public Health Act of New York State. The protocol was approved by University Committee on Animal Resources (UCAR) at University of Rochester (approval number 2007–151). All efforts were made to minimize the suffering of the animals. The physical condition and welfare of the animals was monitored weekly. For subcutaneous tumor injections, mice were anaesthetized by ketamine and xylazine prior to injections. If the animals showed signs of illness (e.g., no grooming, refusal to feed), experiments were terminated and the animals euthanized. Carbon dioxide inhalation was used as the euthanasia method for all animals.

The MA-2 cell line was derived from the A375P cells (ATCC No. CRL-1619) [12]. The WM266-4 [50] and 451LUCS-BR [26] cell lines were received from Wistar Institute, Philadelphia PA, USA. The MeWo cell line was purchased from ATCC (HTB-65). MA-2 cells were cultured in DMEM, 10% FBS, 200 mM glutamine, pen/strep; MeWo cells were cultured in MEM, 10% FBS, 1.5g/L NaHCO_3,_ NEAA (non-essential amino acids), pen/strep; 451LUCS-BR cells were cultured in DMEM, 5% FBS, 200mM glutamine, pen/strep, 1mM SB 590885 (Tocris Bioscience, Bristol, UK). WNT5A and WNT3A recombinant proteins were purchased from R&D Systems. LiCl was purchased from Mallinckrodt Pharmaceuticals.

### Knockdown and overexpression constructs

shRNAs targeting different regions in human *FZD7* were cloned into the lentiviral vector, pLKO.1, as previously described [51]. The targeted sequences are: shFZD7#1 5’–ATCATGGTCATCAGGTACTTG -3’, shFZD7#2 5’–TATGAAGAGGTAGACGAACAG– 3’, shFZD7 #3 5’–TCCTTAAAGTACATCAGGCCG– 3’, shFZD7#4 5’–TCGTTCACTATGGTATCTG– 3’; shFZD7#5 5’- CCGGTGCAGTGGTCACATAAATTT– 3’. The shRNA targeting the Green Fluorescent Protein (5’- CCGGTCAAGCTGACCCTGAAGTTCTT– 3’) was used as the control. Virus was produced in HEK293T cells before infecting melanoma cell lines. Puromycin-resistant clones were selected and amplified. The efficiency of knockdown was analyzed by quantitative RT-PCR.

To express dominant negative JNK in melanoma cells, the pcDNA3 Flag MKK7B2Jnk1a1(APF) construct was obtained from addgene.org. The insert was amplified by PCR and cloned into a modified MSCV-based retroviral expression vector [52]. Virus was produced in HEK293T cells before infecting melanoma cell lines. Hygromycin-resistant clones were selected and amplified.

### Quantitative RT-PCR

Total RNA was extracted from melanoma cell lines using the Qiagen RNeasy Mini kit (Qiagen, Valencia, CA, USA). cDNA was synthesized from 1 μg RNA using the iScript kit (Bio-Rad, Hercules, CA, USA) and served as the template for quantitative PCR (MyiQ2 Two color Real-Time PCR Detection System, Bio-Rad). Primers used were: *FZD7*-forward 5’-GTGCCAACGGCCTGATGTA-3’; *FZD7*-reverse 5’- AGGTGAGAACGGTAAAGAGC-3’; GAPDH-forward 5’-GAAGGTGAAGGTCGGAGTC-3’, GAPDH-reverse 5’-GGAGATGGTGATGGGATTTC-3’; AXIN2-forward 5'- TACACTCCTTATTGGGCGATCA-3’, AXIN2-reverse 5'-TTGGCTACTCGTAAAGTTTTGGT-3’; WNT5A-forward 5'- TTGGTGGTCGCTAGGTATGA-3’, WNT5A-reverse 5'-AGTGGCACAGTTTCTTCTGTC-3’.

### Immunohistochemistry

Mouse lungs were fixed in 10% formalin overnight, processed, paraffin embedded and sectioned. Immunohistochemical analyses were performed according to the standard protocols. Briefly, sections were rehydrated through ethanol series and subjected to antigen retrieval in citrate buffer (pH 6.0). After treatment with H_2_O_2_ and blockage in 5% normal donkey serum, sections were incubated with the antibody against human vimentin (1:400; DAKO, Carpinteria, CA, USA) at 4°C overnight. The signals were detected by biotinylated donkey anti-mouse secondary antibody and amplified with the ABC Kit (Vector Laboratory, Burlingame, CA, USA). Colorimetric development was performed using the VIP or Nova Red Substrate Kit (Vector Laboratory), with hematoxylin as the counterstain. For double labeling, sections were incubated first with the mouse anti-phospho-histone H3 antibody (1:50, BD Transduction Laboratories), at 4°C overnight, followed by secondary antibody detection, signal amplification, and colorimetric development with the Nova Red substrate kit. The sections were then incubated with the mouse anti-human vimentin antibody (1:400) at 4°C overnight, followed by secondary antibody and signal amplification as describe above, but colorimetric development used the VIP substrate kit. Images were taken from five random fields on each section, using the AxioCam ICc1 (Zeiss International) camera. The number of micro metastases in each field was counted and added together as the number of metastases per section.

### *In vitro* treatments, cell fractionation, and western blotting

Melanoma cells were plated in a six-well plate in growth medium. Cells were serum-starved the next day for 24 hrs, then treated with 100 ng/ml WNT5A for 0, 15, 30, 60 and 120 minutes, or with 0 ng/ml, 10 ng/ml, 20 ng/ml and 50 ng/ml of WNT3A for 30 min, or with 0 mM, 10 mM, 20 mM, and 40 mM LiCl for 4 hrs. Cells treated with WNT3A were subject to RNA extraction using the Qiagen RNeasy Mini kit (Qiagen, Valencia, CA, USA), followed by qRT-PCR analyses. Cells treated with WNT5A were lysed in Laemmlie buffer. An equal amount of protein was loaded onto SDS-PAGE and probed with the rabbit anti-pJNK antibody (#9251S, Cell Signaling), rabbit anti-total JNK antibody (sc-571, Santa Cruz Biotechnology), and mouse anti-GAPDH antibody. Signals were amplified by appropriate secondary antibodies and detected by enhanced chemiluminescence detection system (Bio-Rad). Band intensities of pJNK and total JNK were measured by ImageJ software and their ratio under each condition was calculated.

Cells treated with LiCl were either subject to RNA extraction for qRT-PCR analyses, or lysed for fractionation studies. To isolate cytosolic and nuclear fractions, cells were first lysed in buffer A (10 mM HEPES pH 7.9, 10 mM KCl, 10 mM EDTA, 1 mM DTT, 0.4% NP40, protease inhibitors), followed by centrifugation at 15,000 x g for 3 min at 4°C. The supernatant contains the cytosolic fraction. The pellet contains the nuclear fraction and was homogenized in buffer B (20 mM HEPES pH7.9, 400 mM NaCl, 1 mM EDTA, 10% glycerol, 1 mM DTT, protease inhibitors), followed by centrifugation15,000 x g for 5 min at 4°C. The supernatant was the nuclear fraction of the cells. The cytosolic and nuclear fractions were run on SDS-PAGE and probed with the rabbit anti-β-catenin, rabbit anti-lamin A/C, and mouse anti-gamma tubulin antibodies (ThermoFisher). Signals were amplified by appropriate secondary antibodies and detected by enhanced chemiluminescence detection system (Bio-Rad).

### Experimental metastasis assay, time course analyses, and size distribution of metastases

Melanoma cell lines were injected (2X10^4^/mouse for MA-2, 10^5^/mouse for WM266-4, 10^5^/mouse for 451LUCS-BR, and 2.5 X 10^5^/mouse for MeWo) into the tail vein of NSG mice. Mice were sacrificed 4–5 weeks after injection and their lungs were excised and fixed in 10% formalin. The nodules on lung surface were counted under a dissection microscope.

To visualize lung metastases from MA-2 cells 24 hours after injection, cells were pre-labeled with the Cell Tracker Green CMFDA (Life Technologies, Grand Island, NY, USA) at 37°C for 15 min in serum-free DMEM, followed by 1 hr recovery in DMEM containing 10% FBS. The labeled cells were injected into the tail vein of NSG mice and mouse lungs were harvested and frozen in O.C.T (Optimal Cutting Temperature Compound) 24 hrs later. Non-continuous 8-μm cryosections from each lung were fixed with 4% paraformaldehyde and stained with DAPI (4,6-diamidino-2-phenylindole). Images were taken from five random fields on each section, using the Zeiss Axio Imager M2m (Zeiss International, Jena, Germany) and the AxioVision Software 4.8.2 (Zeiss International), and processed by Adobe Photoshop software (Adobe Systems Incorporated, San Jose, CA, USA). The number of green fluorescent cells in each field was counted and added together as the number of fluorescent cells per section.

For other time course studies, melanoma cells were injected into the tail vein of NSG mice. Lungs were harvested at week 1, 2, 3 and 4 for MA-2 cells, or 24 hrs, week 1 and 2 for MeWo cells. They were fixed in 10% formalin, processed, sectioned (8μm) and stained with the mouse anti-human vimentin antibody (1:400; DAKO, Carpinteria, CA, USA). Images were taken as described above, and the number of metastases in each of five fields was counted and added together as the number of metastases per lung section.

To quantitate the difference in metastasis size, the number of cells contained by each metastasis was counted and used to divide metastases into three groups: small, medium, and large. For MA-2 metastases and week 2 MeWo metastases, those containing 1–10 cells were considered small, those containing 11–20 cells were considered medium, and those containing over 20 cells were considered large. The week 1 MeWo metastases were relatively small, so the criteria used to differentiate metastases were slightly different. Those containing 1–5 cells were considered small, those containing 6–10 cells were medium, and those containing over 10 cells were large. A total of 90 metastases from six mice at week 1 and 51 metastases from four mice at week 2 were analyzed for MA-2(shGFP) samples. A total of 88 metastases from 6 mice at week 1 and 137 metastases from six mice at week 2 were analyzed for MA-2(sh*FZD7*) samples. A total of 129 metastases from four mice at week 1 and 71 metastases from three mice at week 2 were analyzed for MeWo(shGFP) samples. A total of 141 metastases from four mice at week 1 and 68 metastases from three mice at week 2 were analyzed for MeWo(FZD7KD) samples. The percentage of metastasis in each size range was calculated and plotted.

### Soft Agar Assay

The soft agar assay was carried out in a 24-well plate. 0.5ml of 0.5% low melting agar (Lonza- Sea Plaque Agarose) in complete culture medium was added in each well to form the bottom layer. 5 x 10^3^ cells mixed with 0.5ml of 0.3% low melting agar in complete culture medium was added on top of it. Three weeks later, images of multiple fields were taken. The number of colonies in each field was counted and the average number of colonies per field calculated.

### Subcutaneous tumor formation, tumor onset, and limiting dilution assay

For subcutaneous tumor formation, 5000 tumor cells were injected into 6–10 weeks old NSG mice underneath their right flank. The mice were sacrificed 4–5 weeks later and tumors were harvested and weighed. For tumor onset assay, 5000 WM266-4 cells were injected subcutaneously into NSG mice. A palpable test was performed once a week to assess tumor growth. Limiting dilution assays were performed by injecting various numbers of melanoma cells subcutaneously into the right flank of NSG mice. Tumor incidence was monitored by palpable tests for up to 12 weeks post injection.

### Data analyses and statistics

For limiting dilution assays, the ELDA (Extreme Limiting Dilution Analysis) method [53] (http://bioinf.wehi.edu.au/software/elda/) was used to measure tumor-initiating frequencies and the difference between samples. For other data analyses, the Graphpad Prism software (www.graphpad.com) was used. Student’s t test was performed for pairwise comparison of data that passed the normality tests, whereas Mann-Whitney test was performed for pairwise comparison of those that did not. Log-rank test was performed to compare the difference in tumor onsets of WM266-4 control and FZD7-knockdown cells.

## Supporting Information

S1 Fig Tumors from WM266-4(sh*FZD7*) cells regained *FZD7* Expression. qRT-PCR was performed to analyze the level of *FZD7* mRNA in WM266-4(shGFP) or WM266-4(sh*FZD7*) tumors. The level of *FZD7* mRNA in the knockdown tumors was no longer lower than that in the shGFP control.
(TIF)

S2 Fig *FZD7* does not affect proliferation of MA-2 cells *in vitro*. Equal numbers of MA-2(shGFP) and MA-2(sh*FZD7*) cells were plated and grown over four days. The number of cells in each well was counted every day.
(TIF)

S3 Fig Expression intensity of WNT ligands in human melanoma metastases. The expression values of *WNT3*, *WNT5A*, *WNT8B*, and *WNT11* in human melanoma metastases (n = 52) were retrieved from the publically available microarray data.
(TIF)

S4 Fig *WNT5A* level in melanoma cell lines, tumor samples, and mouse lung. qRT-PCR was performed to measure the level of *WNT5A* mRNA in MA-2, MeWo, and A375P melanoma cell lines, in the subcutaneous tumors or lung metastases from MA-2 cells, and in mouse lung.
(TIF)
