# Correction: *FRIZZLED7* Is Required for Tumor Inititation and Metastatic Growth of Melanoma Cells

**DOI:** 10.1371/journal.pone.0187388

**Published:** 2017-10-26

**Authors:** Shweta Tiwary, Lei Xu

In the title of this article, the word “Inititation” should be “Initiation.” The correct title is: *FRIZZLED7* Is Required for Tumor Initiation and Metastatic Growth of Melanoma Cells. The correct citation is: Tiwary S, Xu L (2016) *FRIZZLED7* Is Required for Tumor Initiation and Metastatic Growth of Melanoma Cells. PLoS ONE11(1): e0147638. https://doi.org/10.1371/journal.pone.0147638
