# Supplementary figures and images for: FRIZZLED7 Is Required for Tumor Inititation and Metastatic Growth of Melanoma Cells
Source: PLoS One. 2016 Jan 25;11(1):e0147638. doi: 10.1371/journal.pone.0147638 (PMC4726610; doi:10.1371/journal.pone.0147638)

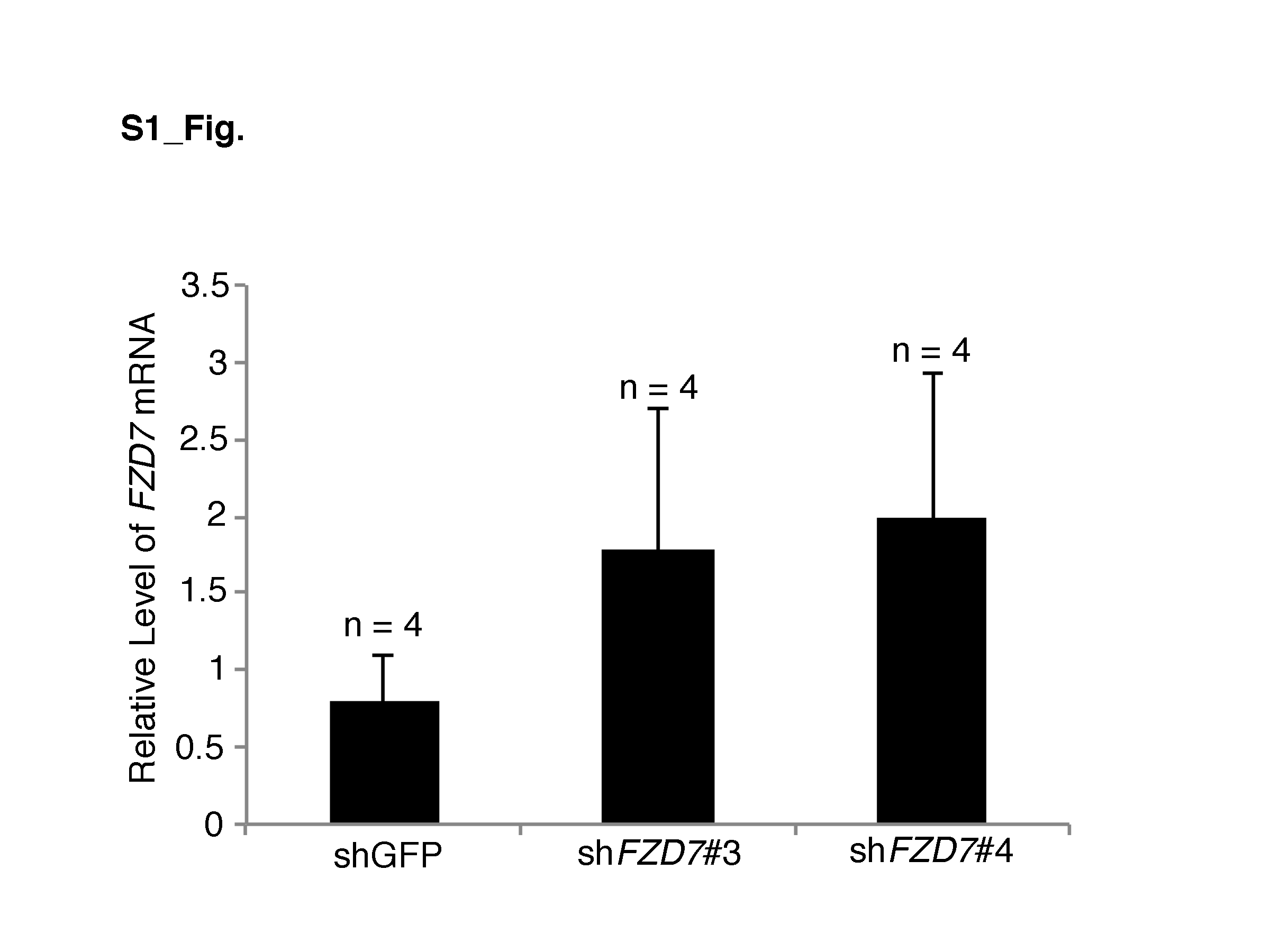

Supplement: S1 Fig — qRT-PCR was performed to analyze the level of FZD7 mRNA in WM266-4(shGFP) or WM266-4(shFZD7) tumors. The level of FZD7 mRNA in the knockdown tumors was no longer lower than that in the shGFP control. (TIF) [file pone.0147638.s001.tif]

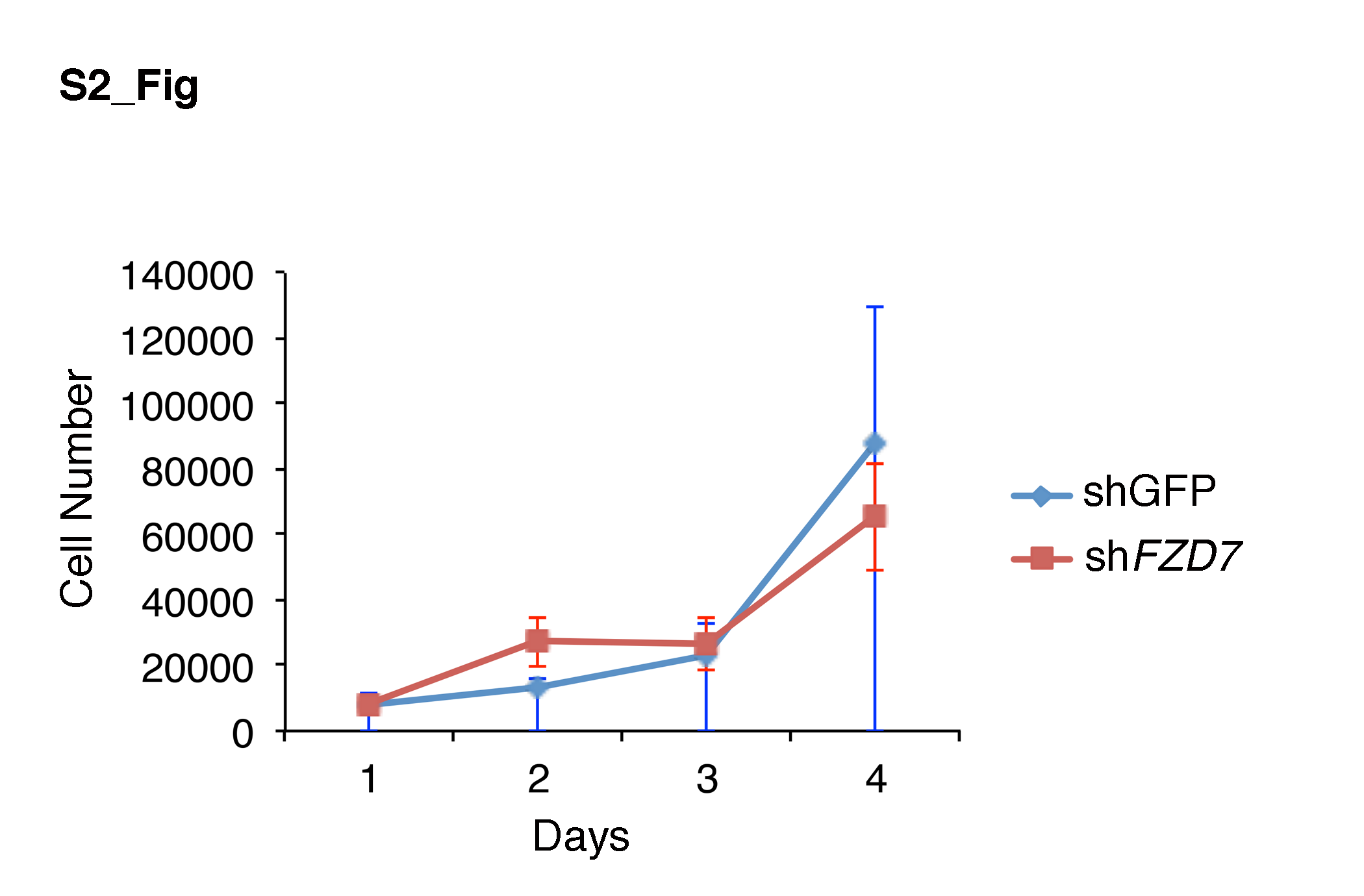

Supplement: S2 Fig — Equal numbers of MA-2(shGFP) and MA-2(shFZD7) cells were plated and grown over four days. The number of cells in each well was counted every day. (TIF) [file pone.0147638.s002.tif]

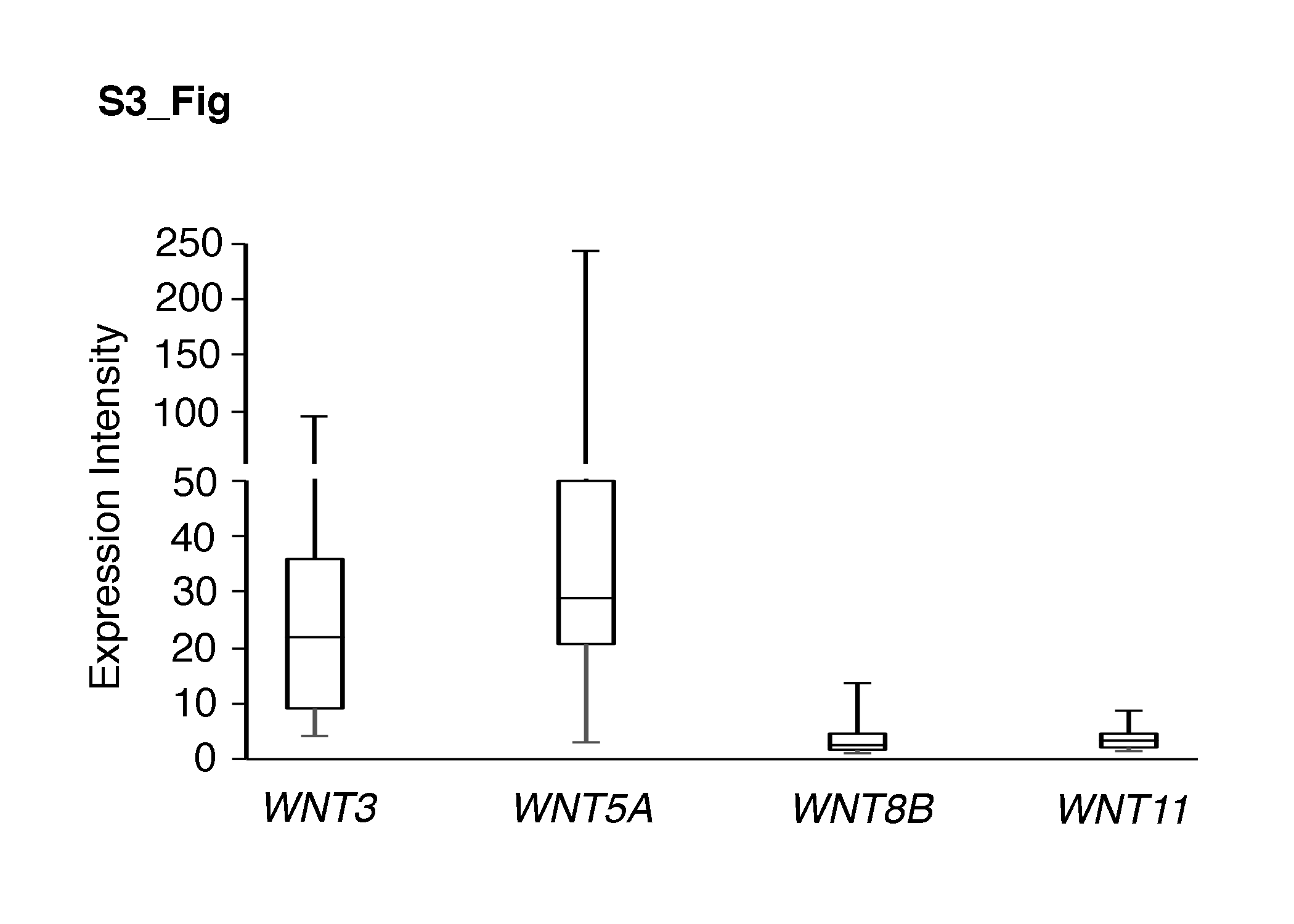

Supplement: S3 Fig — The expression values of WNT3, WNT5A, WNT8B, and WNT11 in human melanoma metastases (n = 52) were retrieved from the publically available microarray data. (TIF) [file pone.0147638.s003.tif]

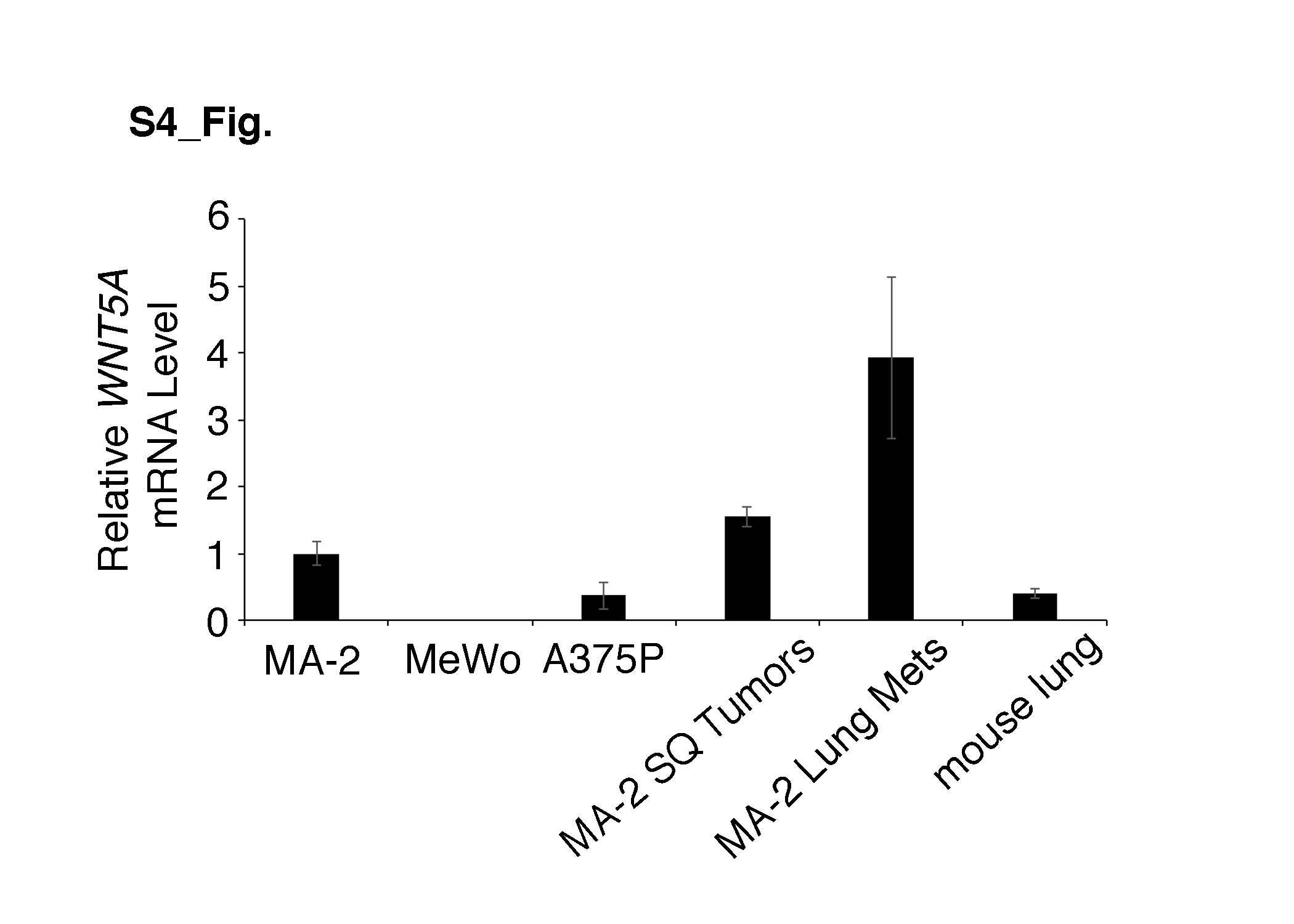

Supplement: S4 Fig — qRT-PCR was performed to measure the level of WNT5A mRNA in MA-2, MeWo, and A375P melanoma cell lines, in the subcutaneous tumors or lung metastases from MA-2 cells, and in mouse lung. (TIF) [file pone.0147638.s004.tif]
